# Around the Hospitals

**Published:** 1894-11-17

**Authors:** 


### Around the Hospitals.

[Notice to the Managers of Institutions.—Special space is now reserved for the insertion of notices of hospital meetings and festivals. The Editor requests that all notices may reach The Hospital Office, 428, Strand, at least one week before the date of each meeting.]

**The New Hospital for Women.**—The work of this hospital is steadily increasing. The question of extending the buildings is under consideration, and a sum for this purpose is accumulating, consisting of a share of £3,131 in the Pfeiffer bequest. The maternity department, which is maintained independently, is also progressing. The convalescent home near Tring has proved most useful. There is also a samaritan fund in connection with the hospital. Paying patients are admitted to the separate wards, nineteen of them being received during the past year. In the general wards 481 patients were treated, and 10,169 persons attended as out-patients. The annual report is clearly and well drawn up, but it is an omission that it does not contain a comparative list of patients admitted year by year. During 1893 the ordinary income amounted to £3,302, and the expenditure to £3,781. The cost of each in-patient was £5 19s. 9d., and each out-patient is estimated as having cost 2s. 2½d., a sum exceeding that which is laid down as an adequate charge for such cases in "Burdett's Hospital and Charities Annual."

**The London Hospital.**—The special claims of this the largest of all our English voluntary hospitals are so apparent as to hardly need reiteration, were it not that the support it receives is all inadequate to its needs. The hospital cannot look to its immediate neighbourhood for maintenance. The inhabitants of its vicinity are at once of the poorest and the most in need of hospital assistance. Yet the Whitechapel poor have and are making efforts to show their appreciation of the opportunities afforded them by contributing what they can towards their hospital. The people's subscription fund reaches £2,000 a year, a praiseworthy total, but one which leaves an enormous margin for help from other sources, for the hospital can only count on a sum of £20,000 to meet the £60,000 income it requires. Its need is not likely to be less, for as the locality becomes more crowded so does sickness increase, and thus the call on the institution is augmented. Last year was the busiest on record, and no less than 10,599 patients were received in the wards, part of that period as many as 717 sick persons being tended in one day. A still greater number attended in the out-patients' department. Besides maintenance and general treatment the calls of so vast an establishment upon its funds are very great. Improvements and additions are constantly necessary to keep up the standard of efficiency. There is a samaritan fund attached to the hospital, which provides assistance of various kinds to needy patients, and secures a visit to a convalescent home to over 1,000 of them annually. Besides this Mr. Peter Reid's munificence secures admission for a further number of patients to his home at Swanley. In spite of this provision there is scope for great extension of this part of the work of the hospital, as it is very difficult for sick persons to regain full health in the crowded and impoverished dwellings of the East-end. Every effort is made to carry on the hospital economically, and the necessities of those treated are carefully inquired into as relief is entirely free. During 1893 the ordinary income amounted to £56,763, and the expenditure to £59,901. The cost per fully occupied bed was £78 4s. 3½d., the cost per patient £5 3s. 2d.

